# Practices and Challenges in Portuguese Early Childhood Intervention: A Descriptive Study

**DOI:** 10.3390/children13020304

**Published:** 2026-02-22

**Authors:** Cristina Costeira, Inês Lopes, Saudade Lopes, Vanda Varela Pedrosa, Susana Custódio, Elisabete Cioga, Cândida G. Silva

**Affiliations:** 1 School of Health Sciences, Polytechnic University of Leiria, Campus 2, Morro do Lena, Alto do Vieiro, Apartado 4137, 2411-901 Leiria, Portugal; ines.lopes@ipleiria.pt (I.L.); saudade.lopes@ipleiria.pt (S.L.); vanda.varela@ipleiria.pt (V.V.P.); susana.custodio@ipleiria.pt (S.C.); elisabete.cioga@ipleiria.pt (E.C.); candida.silva@ipleiria.pt (C.G.S.); 2 Centre for Innovative Care and Health Technology (CiTechCare), Polytechnic University of Leiria, Rua de Santo André—66–68, Campus 5, 2410-541 Leiria, Portugal; 3 The Health Sciences Research Unit: Nursing (UICISA: E), Nursing School of Coimbra (ESEnfC), 3004-011 Coimbra, Portugal

**Keywords:** early intervention, early childhood interventions, infant, child, early developmental intervention, services accessibility, barriers, delivery of care, descriptive study

## Abstract

Background/Objectives: Early Childhood Intervention (ECI) services are critical for supporting children with developmental needs and their families. Despite an established legislative framework, challenges related to accessibility, equity, resources, and standardization of practices persist. This study aimed to describe the perspectives of early intervention professionals in Portugal regarding current barriers, facilitators, and priority areas for improvement within the system. Methods: A descriptive study was conducted involving 82 professionals working in early intervention in Portugal. Data were collected using a survey specifically developed by the research team, grounded in a comprehensive literature review and professional expertise. The instrument was validated through a Delphi Panel with two rounds involving six experts in ECI. Data from open-ended questions were analyzed using content analysis, identifying categories and sub-categories to describe the responses, and descriptive statistics for the closed-ended questions. Results: Professionals highlighted the need to update the National ECI System (SNIPI), improve accessibility, and ensure equitable access to early intervention services. Participants reported limited resources, a lack of standardization in practices, and emphasized the importance of professional training and continuous professional development. The findings also pointed to the urgent need for investment and functional and structural restructuring of early intervention services. Various barriers and facilitators were identified. Conclusions: The study provides valuable insights into the perspectives of early intervention professionals, identifying critical areas for policy improvement, resource allocation, and practice standardization.

## 1. Introduction

The prevalence of developmental disorders in early childhood has been increasing worldwide, posing significant challenges to children’s learning, behavior, and long-term participation in educational and social contexts [1]. In response to this global trend, early childhood intervention (ECI) has been internationally recognized as a key strategy for promoting children’s development and preventing long-term functional limitations, particularly for children with developmental disabilities or at risk of developmental delay [2,3,4]. Contemporary ECI frameworks emphasize a holistic and bioecological perspective, in which child development is understood as the result of dynamic interactions between the child, the family, educational settings, and the broader community [5,6,7,8]. Evidence from community-based programs indicates that fostering participation in natural routines and strengthening the role of caregivers and practitioners within proximal processes are essential for children’s optimal development. Successful intervention depends on achieving a dynamic and adaptive fit between the child’s strengths and needs and the affordances of their immediate environments in which they grow and participate [5,6,7,8].

Globally, leading scientific and professional organizations in early childhood development, such as the World Health Organization [9], the Division for Early Childhood (DEC) of the Council for Exceptional Children [10], Zero to Three Organization [11] and United Nations Children’s Fund (UNICEF) [12,13,14], advocate for family-centered, community-based, and inclusive, early intervention models. These approaches emphasize partnerships with families, collaboration across health and early childhood education systems, and the use of natural learning environments as primary contexts for intervention [5,6,7,9,10,11,12,13,14]. International recommendations consistently highlight the importance of evidence-based practices, including interprofessional collaboration, shared decision-making, and the promotion of children’s participation in meaningful daily routines at home, in early education settings, and within the community [8,9,10,11,12,13,14,15,16,17,18,19,20,21,22]. It also highlights the importance of ensuring that all children, from pregnancy through to 8 years of age, have access to the conditions necessary for healthy and holistic development [14]. Empirical evidence demonstrates that such practices are associated with positive outcomes in children’s cognitive, language, motor, and socio-emotional development, while also strengthening family well-being and empowerment [5,6,7,15,16,17,18,19].

Research grounded in this perspective underscores that the effectiveness of early intervention depends not only on program content, but also on organizational conditions, interprofessional collaboration, and system coherence across sectors [21].

Within this international landscape, the Portuguese ECI system aligns closely with globally recognized principles of high-quality ECI; however, certain practices require reconsideration and further improvement, considering the country’s specific socio-political and cultural contexts.

### 1.1. Research Problem

In Portugal, ECI is coordinated through the National ECI System (SNIPI), defined in Decree-Law N°. 281/2009 [10]. This nationwide, intersectoral system, jointly overseen by the Ministries of Health, Education, and Labour and Social Security [16,17,18,19]. At the national level, SNIPI is jointly overseen by the respective ministries, which are responsible for strategic planning, policy development, and the definition of technical guidelines [16,20]. The regional coordination teams are responsible for adapting and supervising the implementation of national directives, promoting intersectoral collaboration, and supporting the evaluation of service quality across regions [17]. At the local level, the system is operationalized through the Local Intervention Teams (ELIs), which constitute the core of SNIPI’s service delivery [17]. This organizational structure is intended to promote accessibility and ensure equity in the provision of ECI services [17].

SNIPI is grounded in a family-centered and transdisciplinary model to support children from birth to six years of age and their families. Intervention is delivered primarily through ELIs, multidisciplinary teams composed of professionals from the health, education, and social sectors, responsible for early identification, functional assessment, and the implementation of individualized and family-centered support within children’s natural environments [16,17].

Despite the existing legislation, challenges remain regarding accessibility and territorial equity. Regarding SNIPI data from 2024, the number of children awaiting intervention, in relation to the total number of children referenced, a total of 3517 (32%) (at the national level) were waiting for intervention [21]. This finding underscores the need to strengthen policies and strategies that ensure equal opportunities for all children and families, providing equitable access to benefits and support, and highlights the importance of updating national policies to achieve these goals [22].

### 1.2. Contextual and Theoretical Background

Recognizing the need to improve the quality of care provided in Early Childhood Intervention (ECI), policy makers considered it crucial to launch a dedicated research call, allowing scientific evidence to inform and enhance decision-making in this field. Within this context, the PS4Child project was developed, constituting the point of departure for the present study. The project was funded under the Science4Policy 2024 initiative promoted by the Foundation for Science and Technology (FCT), in collaboration with the Centre for Planning and Evaluation of Public Policies (PlanAPP) [23].

Consistent with international initiatives led by scientific organizations dedicated to child development. The project aims to support the development of applied scientific research for public policies, encouraging the production of knowledge and evidence that strengthens the internal policies of the Portuguese Public Administration. PS4Child project’s overall objective is to contribute to informing the development of public policies that strengthen the functioning of the SNIPI and to contribute to the improvement of the accessibility and quality of responses to the individual needs of children and families within the scope of early childhood intervention, ensuring the effectiveness and sustainability of the interventions implemented. Project implementation involved cooperation with national and regional entities.

From a theoretical standpoint, the project is informed by bioecological models of development, which emphasize the central role of proximal processes and the interaction between children, caregivers, professionals, and everyday contexts over time [18,19,20]. By emphasizing partnerships and shared decision-making, current practices have shifted away from older, deficit-focused models. Today, the focus is now on participatory strategies that support child development while strengthening the family unit, a direction strongly supported by both international guidelines and national empirical evidence [22,24]. Developmentally oriented frameworks suggest that translating concepts into practice is highly dependent on organizational conditions, effective teamwork, and system coherence [25,26]. This places ECI professionals at the very heart of service implementation. Their privileged position provides critical insight into how intervention principles are operationalized, revealing specific organizational barriers and resources that shape their work. Consequently, examining professionals’ perspectives is essential for understanding gaps between intended family-centered models and everyday practice, and for informing strategies aimed at improving service quality [15,27,28,29].

In light of this, the present study aims to explore early intervention professionals’ perspectives on the main barriers, facilitators, and priority areas for improvement within Portugal’s National ECI System, contributing to the international discussion on how evidence-based, family-centered early intervention models can be strengthened within complex, intersectoral service systems.

## 2. Materials and Methods

### 2.1. Study Design

This mixed descriptive study was part of a larger project (PS4Child: Contribution to the Development of Public Policies in Early Childhood Intervention), developed in Portugal in 2025. The quantitative component allows for the description of workforce characteristics, service organization, and accessibility patterns, while the qualitative component identifies professionals’ experiences, perceptions, and contextual insights that cannot be captured through structured surveys alone. By combining these complementary methods, the study enables both descriptive reporting and interpretive analysis, aligning directly with the study’s research questions and objectives, and providing a robust framework to examine barriers, facilitators, and opportunities in ECI practice in Portugal. This article was reported according to the Strengthening the Reporting of Observational Studies in Epidemiology (STROBE) statement [30].

### 2.2. Participants

Participants consisted of professionals collaborating with ELIs in Portugal. According to the official SNIPI website [20], the system comprises five regional coordination structures (North, Center, Lisbon and Tagus Valley, Alentejo, and Algarve), encompassing a total of 156 ELIs nationwide. It was not possible to determine the exact number of professionals who received the invitation; consequently, the response rate could not be calculated.

Eligibility criteria included all professionals who: (i) were formally integrated into an ELI, (ii) held an active professional role during the data collection period, and (iii) provided informed consent to participate in the study. Professionals who were not affiliated with ELIs, as well as students or trainees without formal professional responsibilities within the teams, were excluded.

Recruitment was conducted via email invitations sent to ELI coordinators, using publicly available contact information provided on the SNIPI website available during June of 2025. Coordinators were asked to disseminate the survey to all eligible professionals within their teams. Participation was voluntary, anonymous, and no incentives were offered. As participation depended on voluntary response, a non-probabilistic convenience sample was obtained, which may limit representativeness and introduce self-selection bias. Data were collected between June and October 2025.

### 2.3. Survey Development and Description

Since no existing instrument adequately addressed the objectives of this study, a survey was specifically developed based on a comprehensive review of the literature and the professional expertise of the research team. Although the instrument was not originally structured explicitly according to the PPCT dimensions of the bioecological framework, its development was informed by the consideration of multiple ecological levels influencing professional practice. More specifically, the survey included items addressing the respondent (e.g., professional characteristics and individual perceptions), the process (e.g., interactions and practices within intervention settings), and the context (e.g., organizational and systemic conditions), as well as temporal dimensions related to changes in the early intervention system over time.

In a second phase, the questionnaire underwent a content validation process through a Delphi panel composed of experts in the field. This process was conducted in two rounds and involved six professionals from different fields with experience in the SNIPI (three nurses, one speech therapist, one psychologist, and one social worker). Content validity was assessed using the Content Validity Index (CVI). Experts rated each item on a four-point Likert scale regarding relevance. Item-level CVI (I-CVI) was calculated as the proportion of experts rating the item as relevant or very relevant. A minimum I-CVI of 0.78 was considered acceptable. The scale-level CVI (S-CVI/Ave) was computed as the average of I-CVI values across items.

The final questionnaire comprised a total of 45 mandatory items, of which 9 were open-ended questions, and the remaining were closed-ended questions. The instrument was organized into four sections: (i) Sociodemographic data; (ii) Information on professional experience in Early Childhood Intervention (ECI); (iii) Identification of support needs in Early Childhood Intervention; and (iv) Suggestions for improvement in Early Childhood Intervention. The first section included 7 closed-ended questions (e.g., sex, age). The second section comprised 25 items, of which 7 were open-ended questions. The third section consisted of Likert-type questions, with 26 items and five response options (1 = Insufficient; 2 = Sufficient; 3 = Good; 4 = Very good; 0 = Not applicable/I do not know). The fourth section included 3 questions, two of which were open-ended.

### 2.4. Data Analysis

The data analysis combined quantitative and qualitative approaches, selected according to the nature of the variables collected. Quantitative data were analyzed using Microsoft^®^ Excel (v16.103.2). Categorical variables were summarized using absolute and relative frequencies, while continuous variables were reported as mean ± standard deviation or median (interquartile range, Q1–Q3), depending on data distribution and the appropriateness of the mean to represent central tendency. This approach was chosen to ensure a clear and interpretable summary of the sample characteristics and outcomes.

Data from open-ended questions were analyzed using conventional content analysis [31,32]. Two researchers independently reviewed all responses, identified meaningful units, and coded them into categories and subcategories. Discrepancies were resolved through discussion to achieve consensus. Categories and subcategories were subsequently used to organize and describe the responses, allowing for a systematic and transparent interpretation of qualitative data.

These analytic decisions were made to balance rigor and reproducibility, ensuring that both quantitative and qualitative results could be interpreted consistently and compared with similar studies in the field.

### 2.5. Formal and Ethical Procedures

Participation in the study implied informed consent. Participant anonymity was rigorously preserved, and data collection was conducted using a web-based survey platform, which ensured researcher blinding. To maintain data integrity, multiple submissions from the same Internet Protocol (IP) address were automatically blocked through the survey settings. The study protocol received ethical approval from the Ethics Committee of the Polytechnic University of Leiria (No. CE/…/49/2025).

## 3. Results

### 3.1. ELI Professionals’ Profile

A total of 82 professionals collaborating with the ELIs responded to the online survey. The sociodemographic and professional characterization of the participants is presented in Table 1. Participants had a mean (±standard deviation) age of 46.0 ± 9.5 years and were mainly women (*n* = 77; 94%). Most participants were health professionals (*n* = 56; 68%), followed by education professionals (*n* = 20; 25%) and social services professionals (*n* = 6; 7%). Although most participants held a bachelor’s degree (*n* = 47; 57%), 35 (43%) participants held a master’s degree. In terms of professional experience, participants presented a long career in ECI (19.5 ± 10.3 years) and were mostly in permanent contract positions (*n* = 73; 89%).

Although only 45% (*n* = 37) of participants were full-time members of the ELIs, the mean (±standard deviation) working hours per week in the ELIs was 21.8 ± 12.1 (Table 2). Participants provided direct and regular support to a median (Q1–Q3) of 14 (6–20) cases and acted as a mediator on a median (Q1–Q3) of 12 (5–20) cases (Table 2). Interventions were mainly implemented at the child’s home (*n* = 72; 88%) or nursery/kindergarten (*n* = 70; 85%), but also in health units (*n* = 33; 40%) (Table 2).

Regarding the participants’ training in ECI (Table 3), the majority indicated having specialized training in ECI (*n* = 51; 62%). Nonetheless, all participants reported having engaged in different training courses related to ECI, with a total number of hours of training with a median (Q1–Q3) value of 50 (24–106) hours. This training was obtained from SNIPI (*n* = 72; 88%), an education institution (*n* = 70; 85%) or other training institutions (*n* = 33; 40%). In all cases, training included content related to family-centered practices (Table 3). Despite this training background, many participants still reported the need for additional training, in several areas such as early intervention in general (*n* = 30; 37%), intervention strategies tailored to specific conditions [e.g., autism; neurological conditions] (*n* = 13; 16%), family-centered practices as family support (*n* = 13; 16%), monitoring tools (*n* = 10; 12%), teamwork and problem-solving skills (*n* = 4; 5%), legal regulations/laws (*n* = 3; 4%) and communication skills (*n* = 2; 2%).

### 3.2. Characterization of ELIs Activity

#### 3.2.1. Referral, Response Capacity and Transition

Participants reported that most children are referred to ECI by health services, namely general practitioners (*n* = 60; 73%) and pediatricians (*n* = 61; 74%) (Table 4). From educational services, referrals occur mainly from day care centers (*n* = 56; 68%) and kindergarten (*n* = 76; 93%). Other types of referrals include family (*n* = 34; 41%), justice services (*n* = 9; 11%), and social services (*n* = 8; 10%) (Table 4).

The SNIPI referral form is the most common method for referral (*n* = 49; 60%), followed by email (*n* = 31; 38%). The average waiting time for first contact is 1.5 ± 1.8 months, with most of the participants considering that ELIs do not have adequate response capacity to address all referrals (*n* = 65; 79%) (Table 4). Major difficulties reported were related to: (i) Insufficient Human Resources (e.g., *R8: “Shortage of professionals in the team/family members’ refusal, who constantly postpone and cancel interventions”; R22: “Insufficient professionals in the team. Constant turnover of staff for various reasons, such as contract completion”; R37: “Lack of professionals, lack of SPECIFIC training in early intervention for the professionals assigned to the teams”*); and (ii) High workload demanded from professionals (e.g., *R17: “An extensive list of cases eligible for intervention and the difficulty in assigning certain technicians to the team. At the moment, we do not have a psychologist, and the physiotherapist is on maternity leave. We are a team of two full-time teachers, a speech therapist working reduced hours for breastfeeding (25 h), a social worker with 10 h, and a nurse with 6 h per week. It is difficult to provide a quality intervention under these circumstances.”; R10: “Increase in the number of cases while maintaining the same professionals or without increasing the professionals’ working hours; lack of measures for families to have more quality time with their children to promote development and follow-up”*).

Despite the difficulties related to the response capacity, most participants, 80 (92%), considered that there is articulation between the community resources and services when required for intervention or family support, including local government (e.g., *R6: “Municipal Social Services”*), schools, nurseries, kindergarten, health services (hospitals; community health services); social services; court, Child Protection Commission (e.g., *R79: “Hospitals”; R40: “Court; Child Protection Commission; Hospitals”; R59: “education services…”*).

Nonetheless, articulation with community services and resources was reported to be limited by factors such as case specificity, (i) lack of interest (e.g., *R25: “low involvement and interest”*), (ii) inexistence of priority pathways (e.g., *R73: “There should be fast-track pathways for consultations in certain specialties for children with significant difficulties, given the long waiting times for access”*), and (iii) limited time to intervene (e.g., *R61: “Time is needed…”*).

Most of the participants reported preparing a transition plan when a child reached 6 years of age (*n* = 78; 95%), although 5% indicated not to anticipate transitions (Table 4).

#### 3.2.2. Family-Centered Practices

All professionals reported involving the family in most steps of the ECI process (Table S1). Family-centered practices integrated into the child’s early intervention individual plan include family and children needs and family involvement (e.g., *R5:“ The intervention is centered on the concerns of families and on their involvement in the intervention process”; R51: “Gathering the family’s concerns and needs, setting goals and planning strategies to overcome these difficulties, involving the family throughout the entire process with an active role in implementing and evaluating these strategies and in decision-making, developing a collaborative professional–family partnership, and strengthening the family’s autonomy and capacity to care for and promote the child’s healthy development”; R53:“ Child’s routines”; R71: “ all process is planned and developed with families”; R9: “define realistic goals”; R12: “The PIIP belongs to the family, and they are actively involved and participate in its development, in the creation of objectives and activities, and in the strategies to achieve them; in its implementation with the support of the case manager and all parties involved in supporting the child and family; in the evaluation and review of the PIIP, carried out quarterly with the presence of the family and all participants who can take part; in the transition plan incorporated into the PIIP”*). The participants reinforced the idea of family as the central partner in the process (e.g., *R57: “The family is the primary decision-maker”*) and is crucial to the success of the intervention, the family’s preparation (e.g., *R1: “Empower the family to achieve the goals they want to reach according to their concerns*).

To attend to families’ needs more effectively throughout the ECI process, participants considered that the following aspects could be revised: (i) Insufficient material and human resources, highlighting the importance to improve the human resources dotation (e.g., *R8: “More professionals in the teams to reduce the number of cases per worker and to be able to be more present in the lives of the families*”) and material resources available (e.g., *R56: “Greater availability of professionals and material resources”*); and (ii) Clear and well-known workflows in the organization and operation (*R17: “The Social Security agreement should be reviewed and updated so that we can provide a quality response to the families we support. The various entities to which each professional belongs should meet with the regional Subcommittees to understand what Early Intervention entails, so that there is no constant referral to other services that are not part of Early Intervention”*). The participants also identified the importance of continuous professional training and education (e.g., *R82: “We have an excellent legislative framework… however, in my opinion, it is urgent to ensure training for professionals, to guarantee team stability by preventing excessive mobility of staff, and to secure strong investment in early intervention, particularly in reinforcing investment in human resources. It is necessary to provide in-service training accessible to ALL members of the ELI and reflective supervision that ensures the quality of the teams’ interventions”*).

#### 3.2.3. Monitoring of Intervention Processes and Results

The availability and use of tools or instruments for monitoring intervention results in ELIs were reported by 52 (63%) participants. Available tools or instruments included the Family Empowerment Scale (FES), the Denver Developmental Screening Test for Autism, the Schedule of Growing Skills II, as well as other developmental and monitoring tools such as Portage, TALC, and Mary Sheridan’s Developmental Assessment Scale. These instruments support the systematic assessment of child development, family empowerment, and the overall effectiveness of ECI practices, revealing the absence of a consistent, universally used instrument.

Additionally, 51 (61%) participants indicated that the following indicators were used for the continuous monitoring of the intervention process: the Individual Early Intervention Plan (PIPP), which was the most frequently mentioned tool (*n* = 25); family questionnaires; the SGS (*n* = 2); outcome indicators related to case data; and, less frequently, the Schedule of Growing Skills II (*n* = 1). Overall, these findings reveal the use of multiple monitoring tools, limiting comparability across teams, settings, and regions.

### 3.3. Perspectives on ECI in Portugal

#### 3.3.1. Perceived Facilitators and Barriers of Children’s Access to ECI

In the survey, in an open-ended manner, the participants were asked about the main factors that facilitate access to ECI. Their responses were grouped into three main categories: (i) Awareness and Outreach (*n* = 25), (ii) Functioning and Organization (*n* = 50), and (iii) Family Participation (*n* = 7).

**(i) Awareness and Outreach**—Most participants reported that dissemination and availability of information are important to facilitate children’s access to ECI (e.g., *R72: “Greater dissemination among health and education entities, including childcare centers, could facilitate the referral process”*). Knowledge itself was also identified as a crucial element in the process of accessing ECI (e.g., *R80: “Referring professionals should be able to explain the role of the early intervention professional and their involvement with the family”*; *R29: “Outreach to partner organizations and educational settings”*).

This category comprises three subcategories: (a) Information and Knowledge about ELI/SNIPI, (b) Structured Outreach, and (c) Professional Awareness/Sensibility.

**(a) Information and Knowledge** about ELI/SNIPI: Many responses highlight a lack of knowledge or insufficient information about what ELI is, how it works, and when it should be activated. The importance of clear, accessible, and continuous information is emphasized as a factor that facilitates access (e.g., *R49: “Clarification about the functioning/response of ELI and the fact that families feel we are helpful in assisting them with problems of various kinds”*).

**(b) Structured Outreach:** The responses reinforce that outreach efforts should be more structured, systematic, and multi-channel, involving the health, education, and community sectors, with particular focus on professionals responsible for screening (e.g., *R9: “Information from health, education, and family services”*).

**(c) Professional Awareness/Sensibility:** A strong need emerges for ongoing training, awareness-raising, and theoretical alignment to ensure appropriate referrals and consistent practices (e.g., *R10: “Knowledge and awareness to make referrals as early as possible”*).

**(ii) Functioning and Organization**—This dimension was considered of greatest importance by most participants, particularly regarding the ease of referring children and families to ECI. Within the Functioning and Organization category, participants highlighted several subcategories as key factors that facilitate access to ECI.

**(a) Structural Organization of the Early Intervention System:** The overall structure of the system plays a fundamental role in ensuring that services are accessible and coordinated effectively (e.g., *R3: “Effective Coordination among the three ministries”*). Participants recognize the value of a formal and integrated structure, highlighting the coordination among ministries as the foundation of ECI functioning. The centrality of coordinated work across sectors is emphasized as a key element of ECI.

**(b) Rules and Partnerships:** Clear regulations and strong partnerships between institutions are considered essential for a well-functioning system (e.g., *R7: “Cooperation with schools and health centers”*). Participants highlighted the importance of updated regulations, appropriate resources, and guarantees of free and continuous service (e.g., *R38: “ free of charge services”; R39: “Public service available”*).

**(c) Internal Team Organization:** The organization and coordination within professional teams were recognized as important for delivering timely and consistent interventions. Appreciation was noted for the team’s internal functioning, regular meetings, and accessibility of professionals, although limitations due to a lack of human resources were also highlighted (e.g., *R64: “Weekly meetings among the professionals from the different services that make up the team”; R70: “ Transdisciplinary”*).

**(d) Speed and Simplicity of the Referral Process:** Participants emphasized that quick and straightforward referral procedures significantly facilitate access for children and families (e.g., *R25: “Early Identification and Referral”; R33:“ Referrals were facilitated if made by any person involved” and R45: “Communication between sectors (education; social services; health”*). In this subcategory emerged a strong idea of accessibility and universality, with multiple entry points.

**(e) Existence of Clear Protocols:** Participants believe that having well-defined protocols ensures transparency and consistency in practice, supporting both professionals and families in the intervention process. Participants believe that existing clear criteria would ensure equity and efficiency in service delivery, with constant communication serving as a pillar for effective functioning (e.g., *R12: “knowledge of ECI-ELI and the ease of referral and response to the referrer”; R41: “ Communication and networking”*).

**(iii) Family Participation.** In this category, participants highlighted the importance of family availability, proximity, involvement, and understanding of their needs as factors that facilitate access to ECI. Overall, the content reflects a family-centered approach, highlighting the importance of partnership, communication, and responses tailored to the specific needs of each context (e.g., *R52: “Proximity of families”; R54: “Family Availability”; R56: “Parental/Family Involvement”*).

Participants’ perceived barriers to access to ECI are presented in Table 5. The insufficient number of human resources was identified as the main barrier to ECI access (*n* = 61, 74%). In fact, participants identified the need to integrate additional professionals into ELIs—particularly from the health and education sectors—to improve response capacity (Table S2). The lack of information about available services (*n* = 41; 50%), lack of awareness (*n* = 33; 40%), delays in the signaling and referral of cases (*n* = 32; 39%), geographical barriers such as distance and availability of transportation (*n* = 19; 23%), family refusal (*n* = 17; 21%), and difficulties in initiating (*n* = 17; 21%) or maintaining (*n* = 12; 15%) support also represent potentials barriers in children’s access to ECI (Table 5). Only a minority of the participants (*n* = 6; 7%) considered the current eligibility criteria a barrier to ECI access.

#### 3.3.2. Challenges for Professional Activity

The major challenges faced by the participants in their professional activity in ECI are presented in Table 6. The lack of time is the most recognized challenge (*n* = 64; 78%), followed by the lack of precise orientations by SNIPI (*n* = 42; 51%), and the lack of material resources such as physical space in ELIs (*n* = 30; 37%). Organizational and structural aspects, such as the need to work in a multidisciplinary team (*n* = 25; 30%) and the lack of institutional (*n* = 23; 28%) and legislative (*n* = 22; 27%) support, also constitute relevant challenges for their professional activity. The relationship with the family/caregiver was also considered challenging by 19 (23%) participants. On the personal level, professional dissatisfaction (*n* = 12; 15%) and lack of personal motivation (*n* = 8; 10%) were also considered defying aspects by the participants.

In an open-ended question, the high number of cases (*n* = 4; 5%), lack of human resources (*n* = 3; 4%), bureaucratic process associated with the electronic platform for the registration of the intervention plans (*n* = 3; 4%), asymmetries in the income across different professionals’ (*n* = 2; 2%), and a difficult articulation with educational contexts (*n* = 2; 2%) were also perceived as challenges to the professional activity of the participants.

#### 3.3.3. Identification of Support Needs for ECI

When asked whether current policies in ECI contribute to achieving the expected outcomes, responses were distributed as follows: “Yes”: 8 (10%) participants; “Partially”: 54 (66%) participants, and “No”: 20 (24%) participants. To help identify support needs in the organization, functioning and structure of SNIPI, participants rated 26 different features of SNIPI as insufficient (1), sufficient (2), good (3), very good (4) or not applicable/I don’t know (0). Results are presented in Table 7 and Table S3.

Based on the observed median (Q1–Q3) values (Table 7), participants evaluated most aspects (15 out of 26; 58%) as good (3) or very good (4). However, eight (31%) of the rated aspects were considered by most participants as only sufficient (median = 2; highlighted in bold). These include coverage of children with early childhood intervention needs, community resources availability, measures implemented to reduce the geographical barrier, social support provided (financial aid and adapted education structures), support provided by ELIs or other teams (health professionals and social services), number of monthly sessions, articulation between the three Ministries (Education; Health; and Labor, Solidarity and Social Security), and general society perception on early childhood intervention (Table 7 and Table S3). Three aspects were rated as insufficient (median = 1; highlighted in bold and italics): number of professionals involved, number of hours dedicated by professionals and state investment in early childhood intervention. In fact, state investment in early childhood intervention was considered insufficient by 73% of the participants (Table S3).

#### 3.3.4. Improvement Suggestions

Out of the 82 participants, 74 (90%) provided improvement suggestions, which were grouped into four main categories: (i) Structural and organizational updates (*n* = 29; 35%); (ii) Improved working conditions (*n* = 25; 30%); (iii) Educational/training programs (*n* = 8; 10%); and (iv) SNIPI updates (*n* = 7; 9%). One participant (R82) believed that “*We have an excellent legislative framework; however, in my opinion, it is urgent to ensure training for professionals through SNIPI, guarantee team stability by preventing excessive staff mobility, and secure strong investment in early intervention, particularly by increasing investment in human resources. It is necessary to provide in-service training accessible to all members of the ELI and reflective supervision with experienced professionals trained in IPI to ensure the quality of the teams’ interventions*”.

Participants emphasized the importance of improving services and resources, as well as optimizing procedures to make them faster, more efficient, and closer to families and children with needs (e.g., *R66: “Reduction in the number of children supported per mediator, flexibility in working hours”*). Therefore, it is essential to provide better resources, retain professionals, encourage them to develop the area, and standardize care practices, ensuring equity, accessibility, and timeliness (e.g., *R63: “he need for stable contracts for professionals to ensure a continuous and cohesive response”; R51: “Increase the number of professionals in the ELIs to reduce waiting times/lists; appoint a full-time ELI coordinator to meet the requested needs”*).

Professionals also highlighted the importance of investment, particularly in staff training and the provision of necessary materials for care (e.g., *R81: “Strengthening technical and human resources, specialized training, continuous professional development, smaller coverage areas, timely replacement of staff, and faster management response”; R12: “Having a permanent headquarters equipped with all the necessary materials for full operation”*).

## 4. Discussion

This study provides an in-depth understanding of the organization and implementation of SNIPI in everyday practice from the perspective of professionals working in ELIs. By examining professionals’ views on system organization, functioning, access, and working conditions, the findings reveal both strengths and areas for improvement in Portuguese ECI. Although the majority of participants (66%) perceived SNIPI as functioning well, they also indicated that current policies only partially meet existing ECI needs. This suggests that while the structural framework is robust, operational challenges persist, limiting the system’s responsiveness and coverage.

Findings regarding the professional profile of ELI staff participating in the study indicate that ECI in Portugal is predominantly delivered by experienced professionals (mean = 19.5 ± 10.3 years) who are highly qualified. The majority are women (94%) working in health-related fields (56%), with smaller proportions coming from education (25%) and social services (7%). The results indicate that 89% of participants have a permanent contract; however, only 45% are employed full-time in ELIs. This limited level of dedication may compromise the quality and timeliness of care delivery. Although this profile is consistent with international evidence describing the feminization of the workforce in child development and early intervention services [26], it underscores the need to consider workload distribution and dedication as key determinants of service quality.

The multidisciplinary composition of teams, integrating professionals from health, education, and social services sectors, reflects the intersectoral nature of SNIPI and aligns with a systemic and ecological understanding of child development. From a bioecological perspective, this intersectoral composition supports the articulation of multiple microsystems and mesosystems surrounding the child and family, a core principle of Bronfenbrenner’s Process–Person–Context–Time (PPCT) model [33]. However, despite this favorable profile, participants consistently reported organizational constraints, namely a limited capacity to respond to the number of referrals (79%), associated with low human resources; delays in the first contact with families and children following referral (1.5 ± 1.8 months); and high workload demands on professionals. These conditions may undermine the continuity and quality of proximal processes, defined within the PPCT framework as sustained, reciprocal interactions between children, caregivers, and professionals over time. This finding reinforces the existing evidence that the effectiveness of early intervention depends not only on professional competence and dedication, but also on organizational and systemic conditions that enable consistent, relationship-based practice [24]. These findings reinforce international evidence that professional competence alone is insufficient; organizational and systemic conditions are essential to ensure consistent, relationship-based interventions [22,25,27,33].

Participants also identified training in educational programs for ELIs as a priority. Although 62% reported having received training, there was a recognized need for further development of both hard and soft skills in early childhood intervention (ECI). Among professionals who reported having undertaken training in this area, 88% indicated that it was provided by SNIPI, revealing an asymmetric distribution of training opportunities.

This highlights the need to broaden access to both technical and interpersonal skill development, supporting the capacity of professionals to implement high-quality, family-centered practices.

Results also indicate a strong alignment of professionals’ discourse and intentions with family-centered principles. Families are actively involved throughout the intervention process, including needs identification, goal setting, implementation, and evaluation of the Individual Early Intervention Plan (PIIP). The PIIP is described as a collaborative and family-owned tool, grounded in family priorities and daily routines, consistent with international recommendations for family-centered ECI [4,34]. Silva et al. [23] similarly emphasize that effective early intervention in educational contexts is characterized by close collaboration with families, shared decision-making, and interventions embedded in natural routines. From a PPCT perspective, these practices enhance the quality of proximal processes by aligning intervention activities with meaningful everyday interactions. However, professionals also acknowledged that full implementation of family-centered practices is constrained by organizational factors such as limited time, high caseloads, and insufficient resources. These constraints restrict relational proximity, continuity of support, and the flexibility required to adapt interventions to families’ contextual realities. Family availability, influenced by work conditions, economic resources, emotional stress, and health factors, was identified as a critical determinant of engagement. Such contextual variables correspond to person and context characteristics within the PPCT model that modulate the intensity and effectiveness of proximal processes. The literature consistently demonstrates that interventions sensitive to these contextual conditions, and paced according to family rhythms, are more likely to promote sustained engagement and positive developmental outcomes [4,28]. Nonetheless, systemic barriers continue to limit the capacity of professionals to operate these principles consistently [25,26].

Overall, the professionals’ perspectives reveal that SNIPI is grounded in a robust conceptual and legal framework aligned with international standards and bioecological principles. However, its daily operationalization remains constrained by persistent structural and organizational barriers, particularly insufficient human resources, high caseloads, limited available time, perceived inadequacy of public investment in early intervention, and fragilities in intersectoral coordination. These constraints compromise the response capacity across teams and limit the consistent implementation of family-centered practices, helping explain difficulties related to access, continuity, monitoring, and quality assurance across ELIs. National and international evidence underscores that sustainable early intervention systems require long-term investment, workforce stability, continuous professional development, and organizational structures that support reflective practice and intersectoral coordination [14,15,22,26].

The participants identified three principal potential facilitators in the ECI response, such as awareness and outreach, which could be enhanced through greater dissemination and availability of information about the system. This idea is supported by D’Agostino et al. [35] and Milat et al. [36], who emphasize the importance of active dissemination in promoting the implementation of evidence-based practices, which are crucial to quality care. Encompassing families, professionals and even community awareness for the process, everyone can play an active and efficient role in the implementation of protective measures, facilitating access, and work system quality, which are crucial to early interventions. Potential facilitators for implementing faster and more streamlined processes in the ECI context were also identified, including the use of standardized protocols and checklists, as well as ensuring tools to share information among all stakeholders. The use of checklists is considered important for standardizing practices and compiling relevant information to support professionals in decision-making within ECIs [37,38]. Effective communication and information sharing among services and stakeholders are key for coordinated and high-quality early intervention delivery [39]. Ineffective communication has also been reported as a main reason for limitations to collaborative work and effective interventions [39]. The use of universally applied instruments was considered important for collaborative work. In the present study, 63% of participants reported using validated tools to support their daily practice; however, no single tool is used consistently across all ELIs.

The participation of families was identified as a crucial facilitating factor by all survey participants. Accordingly, families should be encouraged to engage actively, and it is essential to provide them with the necessary support. Current international guidelines underscore that effective ECI requires integrated and equitable systems capable of supporting children within their everyday environments while strengthening families and caregivers as central agents of development [26]. In line with these international recommendations, research in ECI has consistently highlighted family-centered approaches as a key framework for promoting both child development and family well-being. Family-centered care is built on a cooperative and collaborative relationship between families and professionals, which is consistently associated with beneficial outcomes for both caregivers and children. As highlighted by Jiménez-Arber et al. [35], these approaches facilitate the meaningful involvement of families in decision-making processes. This active participation promotes feelings of competence, satisfaction, and essential empowerment among those involved. These findings align with complementary evidence showing that family-centered practices enhance parental empowerment, strengthen family functioning, and help parents better understand their children’s developmental needs [40,41]. Störbeck [4] reinforces this as the recommended model for ECI, emphasizing that collaborative partnerships and shared decision-making are key to promoting children’s participation within their everyday, natural environments.

A systematic review by Pacheco-Molero et al. [27] reveals that implementing high-quality family-centered ECI is far from simple. In fact, it faces a complex web of barriers at individual, organizational, and systemic levels. These obstacles range from gaps in professional preparation and rigid working conditions to broader legislative hurdles. Crucially, the authors argue that we cannot resolve these challenges through individual professional effort alone. Instead, they demand sustained, coordinated action across policy frameworks and service organizations. Achieving genuine family-centered intervention, therefore, requires a paradigm shift, one supported by coherent institutional structures and long-term investment. While family-centered approaches provide the guiding principles for early childhood intervention, understanding how these principles are translated into everyday practice requires a theoretical framework capable of capturing the complexity of interactions between children, families, professionals, and service systems.

Participants reported the existence of community articulation resources; however, their utilization was limited by multiple factors, including a lack of interest, the absence of clearly established priority pathways, and insufficient time allocated for intervention. This finding highlights a critical gap in the operationalization of intersectoral collaboration, as community resources are essential for early disability identification and timely access to ECI services. From a bioecological perspective, such resources constitute part of the ecosystem and play a key role in supporting proximal processes between children, families, and professionals [42]. The limited engagement with available community supports not only constrains the effectiveness of individual interventions but also risks exacerbating inequities in service access, particularly for families facing socioeconomic or long-term support barriers. International evidence emphasizes that coordinated community-level supports enhance early detection, facilitate interprofessional collaboration, and contribute to more equitable service delivery [42,43]. Therefore, addressing these barriers—through formalized pathways, structured collaboration mechanisms, and allocation of dedicated time for community engagement—represents a crucial step toward strengthening the systemic capacity of ECI and ensuring consistent, family-centered practices.

Additionally, community resources contribute to family support networks, both formal and informal, which are associated with greater family empowerment and engagement in early intervention services [44]. Such support could be particularly important in assisting families and professionals to anticipate the transition process when children reach six years of age, as only 5% of participants reported planning for this stage in advance.

It is important to note that participants discussed the transition process following the end of formal legal support (six years, rather than the eight years identified by UNICEF) [14]. However, they did not mention the need to initiate ECI. In Portugal, the process begins after birth rather than during the gestational period, as recommended internationally [14], in order to ensure that children have access to the conditions necessary for healthy and holistic development. It is also important to note that, although all participants emphasized the importance of starting interventions as early as possible, they did not specify the exact timing. This may be explained by the perspective that professionals felt they lacked sufficient training and education in this area, which limits broader awareness of the possibilities for early intervention.

At present, compassionate communities have been widely investigated in the context of palliative care [45,46,47], but they may also serve as an important resource in early childhood intervention (ECI). Compassionate communities are defined as initiatives that rely on community engagement, solidarity, and collective ownership to influence sociocultural attitudes, knowledge, behaviors, and perspectives [45]. These communities can play a vital role in early support for children by fostering environments in which families, professionals, and community resources collaborate to promote young children’s development and well-being. Grounded in a strengths-based perspective, compassionate community approaches recognize that children and families benefit not only from formal early intervention services but also from informal support networks, including peers, neighbors, and local organizations, which provide social, emotional, and practical assistance [45]. In this way, community engagement and mutual support could become essential components of comprehensive early support systems, reinforcing the idea that nurturing environments extend beyond clinical settings to include the broader social fabric in which families live.

### 4.1. Contributions to the Development of ECI

This study advances knowledge by providing empirical insights into the interplay between organizational structures, workforce characteristics, community resources, and implementation challenges. It offers both descriptive and interpretive evidence that complements existing national studies and contributes to the broader international discourse on effective ECI practices. Specifically, it identifies organizational barriers that limit the consistent implementation of family-centered practices, including high workloads, insufficient human resources, and constraints in service organization. These findings align with recent international evidence, reinforcing the need for organizational-level interventions within ECI systems to ensure quality and sustainability [26,27].

The study further provides applied contributions by highlighting that improvements in ECI quality depend on organizational strategies, such as workforce planning, workload monitoring, and adequate team staffing, in line with international recommendations [9,10,11,12,13,14,26].

Additionally, the findings support consideration of more flexible service delivery models. International evidence indicates that tele-intervention can be effectively applied across all stages of early childhood services—from prevention and assessment to intervention—regardless of the service setting. When appropriately integrated, tele-intervention may expand access, enhance continuity of care, and improve service quality, particularly for families living in rural or remote areas with limited access to urban-based services [35].

### 4.2. Study Strengths and Limitations

This study presents several strengths. The data collection instruments used were specifically developed by the research team and validated with professionals and experts in the field of early intervention, ensuring relevance and content validity. The study includes a diverse sample of professionals from various regions of Portugal, providing a broad perspective on the current challenges and needs within early intervention services. Additionally, the research team itself presents a multidisciplinary composition, bringing together expertise from different fields, which enriched the analysis despite their primary academic commitments.

Nonetheless, some limitations must be acknowledged. The sample size is not fully representative of the total number of active professionals in ELIs, as the total number of professionals involved was neither up-to-date nor shared. This recruitment method may have introduced bias in both participation and the study results, as contacting coordinators via publicly available email addresses on the SNIPI website did not guarantee that all eligible professionals received the invitation, particularly given staff turnover within the teams. Consequently, the sample may overrepresent professionals who were more accessible or engaged, limiting the generalizability of the findings.

Additionally, the voices of families, considered crucial for identifying barriers, facilitators, and specific improvement suggestions, are not represented. Although a parallel study was designed to capture family perspectives, and ELIs were invited to disseminate the survey to families and caregivers, no responses were obtained. Finally, as a descriptive study, the findings are inherently limited in terms of generalizability and causal inference. This methodological choice was deliberate, reflecting the study’s primary aim of documenting and characterizing professionals’ perspectives rather than testing hypotheses or establishing causal relationships. Despite these limitations, the study provides valuable insights into current practices and challenges, highlighting critical areas for future development, investment, and policy improvement in ECI services.

### 4.3. Future Contributions

Based on the findings of this study, it is recommended that SNIPI be updated to reflect current evidence and best practices. Efforts should be made to improve accessibility, equity, and standardization in early intervention services, ensuring timely and appropriate support for all children and families in need. Increasing both human and material resources is essential to reducing waiting times and enhancing the quality of interventions. The standardization of practices across teams would promote consistency and a high standard of care. In addition, investing in professional training, including specialized and continuous education for all staff, is crucial. Functional and structural restructuring of the system is also needed to improve efficiency, coordination, and service delivery. Addressing current barriers while reinforcing facilitators identified by professionals on the front line will further strengthen the system. Continuous monitoring and evaluation of these initiatives are necessary to guide ongoing improvements in early intervention services.

## 5. Conclusions

This study highlights several critical needs and challenges within the current system. Participants emphasized the urgency of updating SNIPI, improving accessibility, ensuring equity, and facilitating access to services. Limited resources, a lack of standardization in practices, and the need for professional training were consistently reported. Additionally, they stressed the necessity of substantial investment along with functional and structural restructuring of the system and current practices. The study also identified multiple barriers and facilitating factors affecting early intervention in Portugal. Despite the limitations, the results highlight valuable insights for informing future policy, resource allocation, and practice improvements in early intervention services in Portugal.

## 6. Patents

This work was developed within the scope of the Science4Policy 2024 (S4P-24) competition, the Science for Public Policy Studies contest, an initiative of the Centre for Planning and Public Policy Evaluation (PlanAPP) in partnership with the Foundation for Science and Technology, I. P., and funded by the Recovery and Resilience Plan.

## Figures and Tables

**Table 1:** Participants’ sociodemographic and professional characteristics (*n* = 82).

Variable	Values
Age (years), M ± SD	46.0 ± 9.5
Sex, *n* (%)	
Female	77 (94%)
Male	5 (6%)
Education level, *n* (%)	
Bachelor’s	47 (57%)
Master’s	35 (43%)
Professional area, *n* (%)	
Health	56 (68%)
Education	20 (25%)
Social Services	6 (7%)
Professional experience in ECI (years), M ± SD	19.5 ± 10.3
Employment contract, *n* (%)	
Permanent contract	73 (89%)
Other	9 (11%)

Abbreviations: M—Mean; SD—Standard deviation.

**Table 2:** Participants’ experience in local intervention teams (ELIs) (*n* = 82).

Variable	Values
Full-time members [Yes], *n* (%)	37 (45%)
Working hours per week, M ± SD	21.8 ± 12.1
Number of cases…, Md (Q1–Q3)	
…providing direct and regular support	14 (6–20)
…acting as mediator	12 (5–20)
Places of intervention ^1^, *n* (%)	
Home	72 (88%)
Nursery/Kindergarten	70 (85%)
Health Unit	33 (40%)

^1^ Multiple choice question. Abbreviations: M—Mean; SD—Standard deviation; Md—Median; Q1—1st quartile; Q3—3rd quartile.

**Table 3:** Participants’ training and training needs in ECI (*n* = 82).

Variable	Values
Specialized training in ECI [Yes], *n* (%)	51 (62%)
Hours of training in ECI, Md (Q1–Q3)	50 (24–106)
Training institution ^1^, *n* (%)	
SNIPI	72 (88%)
Education institution	70 (85%)
Other institution	33 (40%)
Training includes family-centered practices [Yes], *n* (%)	82 (100%)
Training needs [Yes], *n* (%)	82 (100%)

^1^ Multiple choice question. Abbreviations: Md—Median; Q1—1st quartile; Q3—3rd quartile; SNIPI—National Early Childhood Intervention System.

**Table 4:** Referral services, response capacity and transition from ELIs (*n* = 82).

Variable	Values
Referral service ^1^, *n* (%)	
General Practice	60 (73%)
Pediatrics	61 (74%)
Neurology	8 (10%)
Physiatry	3 (4%)
Child psychiatry	16 (20%)
Other health professionals	3 (4%)
Nursery (4–12 months)	7 (9%)
Day care (12–36 months)	56 (68%)
Kindergarten (>36 months)	76 (93%)
Social Services	8 (10%)
Justice Services	9 (11%)
Family	34 (41%)
Referral method, n (%)	
SNIPI referral form	49 (60%)
Email	31 (38%)
Information System	2 (2%)
Average waiting time for first contact (months), M ± SD	1.5 ± 1.8
ELI has response capacity for all referrals, *n* (%)	
Yes	17 (21%)
No	65 (79%)
Transition plan for 6-year-old children [Yes], *n* (%)	78 (95%)

^1^ Multiple choice question. Abbreviations: M—Mean; SD—Standard deviation; SNIPI—National Early Childhood Intervention System.

**Table 5:** Barriers to accessing ECI (*n* = 82).

Barriers	*n* (%)
Insufficient human resources	61 (74%)
Lack of information about available services	41 (50%)
Lack of awareness	33 (40%)
Delays in signaling and referral	32 (39%)
Geographical barriers (distance, transportation, …)	19 (23%)
Family refusal	17 (21%)
Difficulties in initiating support	17 (21%)
Difficulties in maintaining support	12 (15%)
Eligibility restrictions for service access	6 (7%)

**Table 6:** Challenges hindering professional activity in ECI (*n* = 82).

Challenges	*n* (%)
Lack of time	64 (78%)
Lack of precise orientations by SNIPI	42 (51%)
Lack of material resources	30 (37%)
Multidisciplinary collaborations	25 (30%)
Lack of institutional support	23 (28%)
Lack of legislative support	22 (27%)
Relationship with family/caregiver	19 (23%)
Professional unsatisfaction	12 (15%)
Lack of personal motivation	8 (10%)

Abbreviations: SNIPI—National Early Childhood Intervention System.

**Table 7:** Evaluation of support needs (*n* = 82).

Feature	Md (Q1–Q3)
Child and family access to ELI support	**3 (2–4)**
Coverage of children with early childhood intervention needs	**2 (1–3)**
Average waiting time for the first contact	**3 (2–4)**
Collaboration between ECI technicians and family/caregivers	**3 (3–4)**
Process of sharing information between the family and the multidisciplinary team	**3 (3–4)**
ELI support to the family	**3 (3–4)**
Indicators for continuous monitoring/evaluation of the intervention process	**3 (2–3)**
Family involvement in the process	**3 (2–3)**
Number of professionals involved	**1 (1–3)**
Number of hours dedicated by professionals	**1 (1–2)**
Community resources availability	**2 (1–3)**
Measures implemented to reduce the geographical barrier	**2 (1–3)**
Social support provided (financial aid and adapted education structures)	**2 (1–3)**
Support provided by ELIs or other teams (health professionals and social services)	**2 (1–3)**
Educational support provided by ELI or its collaborators to children and families	**3 (1–3)**
Professionals’ involvement in the development of integrated child support plans and integrated child and family support plans	**3 (3–4)**
Professionals’ satisfaction with how the processes are managed	**3 (2–3)**
Flexibility in the articulation between partners	**3 (2–4)**
Number of monthly sessions	**2 (1–3)**
Result of the investment for the child/family	**3 (2–3)**
Continuity of the monitoring process	**3 (2–3)**
Contact/visit log system	**3 (2–3)**
Articulation between the three Ministries (Education; Health; and Labor, Solidarity and Social Security)	**2 (1–3)**
Societal perception of early childhood intervention	**2 (1–3)**
Parents’ perception of early childhood intervention	**3 (2–3)**
State investment in early childhood intervention	**1 (1–1)**

Abbreviations: Md—Median; Q1—1st quartile; Q3—3rd quartile; 1—Insufficient; 2—Sufficient; 3—Good; 4—Very good.

## Data Availability

The data used in this study are contained within the article or Supplementary Material.

## Supplementary Materials

The following supporting information can be downloaded at: https://www.mdpi.com/article/10.3390/children13020304/s1.

## Abbreviations

The following abbreviations are used in this manuscript:

ECI	Early childhood intervention
SNIPI	National Early Childhood Intervention System
PlanAPP	Centre for Planning and Public Policy Evaluation
ELIs	Local intervention teams
PIIP	Individual Early Intervention Plan
PS4Child	Contribution to the Development of Public Policies in Early Childhood Intervention (Project)

## References

[1] Kim S. (2022). Worldwide national intervention of developmental screening programs in infant and early childhood. Clin. Exp. Pediatr..

[2] Hoffmann S., Sander L., Wachtler B., Blume M., Schneider S., Herke M. (2022). Moderating or mediating effects of family characteristics on socioeconomic inequalities in child health in high-income countries: A scoping review. BMC Public Health.

[3] Almeida A., Pereira A.P., Serrano A. (2024). First years: Development, early childhood intervention, and inclusion. Child Stud..

[4] Störbeck C. (2024). Early childhood development is not enough: In defense of children with developmental delays and disabilities and their right to family-centered early childhood intervention (in the Global South). Children.

[5] Gündoğmuş E., Bumin G., Yalçın S.S. (2024). Effect of early intervention on developmental domains and parent–child interaction among children with developmental delay: A randomized controlled study. Am. J. Occup. Ther..

[6] Kumar S.V., Narayan S., Malo P.K., Bhaskarapillai B., Thippeswamy H., Desai G. (2022). A systematic review and meta-analysis of early childhood intervention programs for developmental difficulties in low- and middle-income countries. Asian J. Psychiatry.

[7] Gómez-Cotilla R., López-De-Uralde-Selva M.Á., Valero-Aguayo L. (2024). Efficacy of early intervention programmes: Systematic review and meta-analysis. Psicol. Educ..

[8] Tollan K., Jezrawi R., Underwood K., Janus M. (2023). A review on early intervention systems. Curr. Dev. Disord. Rep..

[9] World Health Organization (2025). Early Childhood Development [Internet].

[10] Division for Early Childhood (DEC) of the Council for Exceptional Children (2025). Division for Early Childhood of the Council for Exceptional Children [Internet].

[11] Zero to Three (2025). Early Development: Building a Strong Foundation [Internet].

[12] United Nations Children’s Fund (UNICEF) (2023). Early Childhood Intervention and Inclusion [Internet].

[13] United Nations Children’s Fund (UNICEF) (2025). Early Childhood Development [Internet].

[14] United Nations Children’s Fund (UNICEF) (2023). Early Childhood Development: UNICEF Vision for Every Child [Internet].

[15] Venancio S.I., de Bortoli M.C., de Freitas Oliveira C., Luquine C.D., Setti C., Melo D.S. (2023). Implementation research in the area of early childhood: Scoping review. Acta Paul. Enferm..

[16] Decreto-Lei n.º 281/2009, de 6 de Outubro. Diário da República. https://diariodarepublica.pt/dr/detalhe/decreto-lei/281-2009-491397.

[17] Sistema Nacional de Intervenção Precoce na Infância. Quem Somos. SNIPI. (n.d.). https://snipi.gov.pt/quem-somos#no-back.

[18] Portugal Resolução do Conselho de Ministros n.º 3/2023, de 10 de Janeiro. Diário da República. https://diariodarepublica.pt/dr/detalhe/resolucao-do-conselho-de-ministros/3-2023-198506031.

[19] Direção-Geral da Saúde Norma n.º 015/2015—Programa Nacional de Saúde Infantil e Juvenil. https://www.dgs.pt/normas-orientacoes-e-informacoes/normas-e-circulares-normativas/norma-n-0152015-de-31072015.aspx.

[20] Sistema Nacional de Intervenção Precoce na Infância. Orientação Técnica n.º 2/2018/SNIPI-CC, de 7 de Dezembro de 2018. https://aemfp.pt/docs/OT_n%C2%BA2_2018_Articulacoo_SNIPI-EI.pdf.

[21] Sistema Nacional de Intervenção Precoce na Infância (SNIPI) (2024). Relatório de Atividade SNIPI 2023. SNIPI. https://snipi.gov.pt/sites/default/files/2024-09/Relat%C3%B3rio%20de%20Atividade%20SNIPI%202023.pdf.

[22] Franco V., Melo M., Santos G., Apolónio A., Amaral L. (2017). A national early intervention system as a strategy to promote inclusion and academic achievement in Portugal. Front. Psychol..

[23] Fundação para a Ciência e a Tecnologia Science4Policy 2024 (S4P-24): Concurso de Estudos de Ciência para as Políticas Públicas. https://www.fct.pt/concursos/science4policy-2024-s4p-24-concurso-de-estudos-de-ciencia-para-as-politicas-publicas.

[24] Silva M.I.A.F., Carvalho C.I., Serrano A.M., Della Barba P.C.D.S. (2025). Intervenção precoce na infância em Portugal: Contexto da creche e jardim de infância. Zero-a-Seis.

[25] Guralnick M.J. (2020). Applying the developmental systems approach to inclusive community-based early intervention programs: Process and practice. Infants Young Child..

[26] World Health Organization (2023). Global Report on Children with Developmental Disabilities.

[27] Pacheco-Molero M., Morales-Murillo C., León-Estrada I., Gutiérrez-Ortega M. (2024). Barriers perceived by professionals in family-centered early intervention services: A systematic review. Int. J. Early Child..

[28] Tong P., An I.S. (2023). Review of studies applying Bronfenbrenner’s bioecological theory in international and intercultural education research. Front. Psychol..

[29] McLinden M., Lynch P., Soni A., Artiles A., Kholowa F., Kamchedzera E. (2018). Supporting children with disabilities in low- and middle-income countries through a bioecological systems perspective. Int. J. Early Child..

[30] von Elm E., Altman D.G., Egger M., Pocock S.J., Gøtzsche P.C., Vandenbroucke J.P. (2008). The Strengthening the Reporting of Observational Studies in Epidemiology (STROBE)statement: Guidelines for reporting observational studies. J. Clin. Epidemiol..

[31] Bardin L. (2011). Análise de Conteúdo.

[32] Valle P.R.D., Ferreira J.D.L. (2025). Content analysis in the perspective of Bardin: Contributions and limitations for qualitative research in education. Educ. Rev..

[33] Bronfenbrenner U., Morris P.A., Lerner R.M. (2007). The bioecological model of human development. Handbook of Child Psychology.

[34] Pereira A.P.S., Serrano A.M. (2014). Early intervention in Portugal: Study of professionals’ perceptions. J. Fam. Soc. Work.

[35] Jimenez-Arberas E., Casais-Suarez Y., Fernandez-Mendez A., Menendez-Espina S., Rodriguez-Menendez S., Llosa J.A. (2024). Evidence-based implementation of the family-centered model and tele-intervention in early childhood services. Healthcare.

[36] D’Agostino S.R., Frost K.M., Pickard K. (2024). Considerations for effective dissemination of evidence-based early intervention approaches. Autism.

[37] O’Connor S., McGilloway S., Hickey G., Barwick M. (2021). Disseminating early years research: An illustrative case study. J. Child. Serv..

[38] Dunst C.J. (2017). Research foundations for evidence-informed early childhood intervention performance checklists. Educ. Sci..

[39] Albuquerque J., Aguiar C., Magalhães E. (2020). The collaboration between early childhood intervention and child protection systems. Child Youth Serv. Rev..

[40] Dick C.C., Ibañez L.V., DesChamps T.D., Attar S.M., Stone W.L. (2022). Perceptions of family-centered care across service delivery systems. J. Autism Dev. Disord..

[41] Frugone-Jaramillo M., Gràcia M. (2023). Family-centered approach in early childhood intervention of a vulnerable population. Front. Psychol..

[42] LoCasale-Crouch J., Lenahan T., Shea Z., Whittaker J., Zhang Y., Duyile B. (2025). Community resources and early disability identification. Prev. Sci..

[43] World Health Organization (2018). Nurturing Care for Early Childhood Development: A Framework for Helping Children Survive and Thrive to Transform Health and Human Potential.

[44] Martínez-Rico G., Simón C., Cañadas M., McWilliam R. (2022). Support networks and family empowerment in early intervention. Int. J. Environ. Res. Public Health.

[45] Dumont K., Marcoux I., Warren É., Alem F., Alvar B., Ballu G. (2022). How compassionate communities are implemented and evaluated in practice. BMC Palliat. Care.

[46] Quintiens B., Vanderstichelen S., Abel J., Kellehear A. (2025). Compassionate communities. Research Handbook on End-of-Life Care and Society.

[47] Thelly A.S., Verginia A.S., McNamara-Goodger K., Ellis P. (2024). Building and sustaining compassionate communities with children. Children’s Palliative Nursing Care.

