# Supplementary material for: Practices and Challenges in Portuguese Early Childhood Intervention: A Descriptive Study
Source: Children (Basel). 2026 Feb 22;13(2):304. doi: 10.3390/children13020304 (PMC12940036; doi:10.3390/children13020304)
Supplement: Supplementary file 1 [file children-13-00304-s001.zip › children-4123578-supplementary.pdf]

children

MDPI

Supplementary Material

Table S1. Families' involvement in the different steps of the ECI process (n=82).

Steps of the ECI process	n (%)
First contact	82 (100%)
Child and family evaluation	79 (96%)
Intervention planning	79 (96%)
Intervention implementation	75 (91%)
Objectives evaluation and reevaluation	80 (98%)
Transition plan	74 (90%)
Process closing	76 (93%)

Table S2. Professionals needed to improve ECI's response capacity (n=82).

Needed professionals	n (%)
Nurse	35 (43%)
Doctor	51 (62%)
Speech and language therapeutist	55 (67%)
Occupational therapeutist	57 (70%)
Physiotherapeutist	44 (54%)
Phychologist	47 (57%)
Social Service technician	29 (35%)
Teacher / kindergarten teacher	40 (49%)
Social Educator	20 (24%)
Psychometrician	3 (4%)
Nutritionist	1 (1%)

Table S3. Evaluation of support needs (n=82).

Feature	Md (Q1-Q3)	Md (Q1-Q3)[§]
**Child and family access to ELIs support**	**3 (2-4)**	**3 (2-4)**
1 – Insufficient	13 (16%)	
2 – Sufficient	9 (11%)	
3 – Good	33 (40%)	
4 – Very good	25 (30%)	
0 – Not applicable / I don't know	2 (2%)	
**Coverage of children with early childhood intervention needs**	**2 (1-3)**	**2 (1-3)**
1 – Insufficient	32 (39%)	
2 – Sufficient	18 (22%)	
3 – Good	19 (23%)	
4 – Very good	12 (15%)	
0 – Not applicable / I don't know	1 (1%)	
**Average waiting time for the first contact**	**3 (2-4)**	**3 (2-4)**
1 – Insufficient	16 (20%)	
2 – Sufficient	18 (22%)	
3 – Good	15 (18%)	
4 – Very good	30 (37%)	
0 – Not applicable / I don't know	3 (4%)	

**Collaboration between ECI technicians and family/caregivers**	**3 (3-4)**	**3 (3-4)**
1 – Insufficient	4 (5%)	
2 – Sufficient	8 (10%)	
3 – Good	31 (38%)	
4 – Very good	39 (48%)	
0 – Not applicable / I don't know	--	
**Process of sharing information between the family and the multidisciplinary team**	**3 (3-4)**	**3 (3-4)**
1 – Insufficient	3 (4%)	
2 – Sufficient	13 (16%)	
3 – Good	33 (40%)	
4 – Very good	32 (39%)	
0 – Not applicable / I don't know	1 (1%)	
**ELI's support to the family**	**3 (3-4)**	**3 (3-4)**
1 – Insufficient	9 (11%)	
2 – Sufficient	4 (5%)	
3 – Good	41 (50%)	
4 – Very good	28 (34%)	
0 – Not applicable / I don't know	--	
**Indicators for continuous monitoring/evaluation of the intervention process**	**3 (2-3)**	**3 (2-3)**
1 – Insufficient	11 (13%)	
2 – Sufficient	19 (23%)	
3 – Good	38 (46%)	
4 – Very good	8 (10%)	
0 – Not applicable / I don't know	6 (7%)	
**Family involvement in the process**	**3 (2-3)**	**3 (2-3)**
1 – Insufficient	7 (9%)	
2 – Sufficient	21 (26%)	
3 – Good	43 (52%)	
4 – Very good	11 (13%)	
0 – Not applicable / I don't know	--	
Number of professionals involved	**1 (1-3)**	**1 (1-3)**
1 – Insufficient	44 (54%)	
2 – Sufficient	11 (13%)	
3 – Good	22 (27%)	
4 – Very good	3 (4%)	
0 – Not applicable / I don't know	1 (1%)	
Number of hours dedicated by professionals	**1 (1-2)**	**1 (1-2)**
1 – Insufficient	47 (57%)	
2 – Sufficient	16 (20%)	
3 – Good	11 (13%)	
4 – Very good	7 (9%)	
0 – Not applicable / I don't know	1 (1%)	
**Community resources availability**	**2 (1-3)**	**2 (1-3)**
1 – Insufficient	33 (40%)	
2 – Sufficient	25 (30%)	
3 – Good	20 (24%)	
4 – Very good	3 (4%)	
0 – Not applicable / I don't know	1 (1%)	
**Measures implemented to reduce the geographical barrier**	**2 (1-3)**	**2 (1-3)**
1 – Insufficient	26 (32%)	
2 – Sufficient	24 (29%)	

3 – Good	17 (21%)	
4 – Very good	6 (7%)	
0 – Not applicable / I don't know	9 (11%)	
**Social support provided (financial aid and adapted education structures)**	**2 (1-3)**	**2 (1-2)**
1 – Insufficient	36 (44%)	
2 – Sufficient	25 (30%)	
3 – Good	15 (18%)	
4 – Very good	4 (5%)	
0 – Not applicable / I don't know	2 (2%)	
**Support provided by ELIs or other teams (health professionals and social services)**	**2 (1-3)**	**2 (1-3)**
1 – Insufficient	29 (35%)	
2 – Sufficient	19 (23%)	
3 – Good	24 (29%)	
4 – Very good	9 (11%)	
0 – Not applicable / I don't know	1 (1%)	
**Educational support provided by ELI or its collaborators to children and families**	**3 (1-3)**	**3 (1-3)**
1 – Insufficient	21 (26%)	
2 – Sufficient	19 (23%)	
3 – Good	25 (30%)	
4 – Very good	16 (20%)	
0 – Not applicable / I don't know	1 (1%)	
**Professionals' involvement in the development of integrated child support plans and integrated child and family support plans**	**3 (3-4)**	**3 (3-4)**
1 – Insufficient	5 (6%)	
2 – Sufficient	13 (16%)	
3 – Good	38 (46%)	
4 – Very good	23 (28%)	
0 – Not applicable / I don't know	3 (4%)	
**Professionals' satisfaction with how the processes are managed**	**3 (2-3)**	**3 (2-3)**
1 – Insufficient	11 (13%)	
2 – Sufficient	20 (24%)	
3 – Good	35 (43%)	
4 – Very good	1 (1%)	
0 – Not applicable / I don't know	3 (4%)	
**Flexibility in the articulation between partners**	**3 (2-4)**	**3 (2-4)**
1 – Insufficient	8 (10%)	
2 – Sufficient	19 (23%)	
3 – Good	32 (39%)	
4 – Very good	23 (28%)	
0 – Not applicable / I don't know	--	
**Number of monthly sessions**	**2 (1-3)**	**2 (1-3)**
1 – Insufficient	26 (32%)	
2 – Sufficient	22 (27%)	
3 – Good	19 (23%)	
4 – Very good	10 (12%)	
0 – Not applicable / I don't know	5 (6%)	
**Result of the investment for the child/family**	**3 (2-3)**	**3 (2-3)**
1 – Insufficient	12 (15%)	
2 – Sufficient	18 (22%)	
3 – Good	35 (43%)	

4 – Very good	16 (20%)	
0 – Not applicable / I don't know	1 (1%)	
**Continuity of the monitoring process**	**3 (2-3)**	**3 (2-3)**
1 – Insufficient	15 (18%)	
2 – Sufficient	18 (22%)	
3 – Good	33 (40%)	
4 – Very good	14 (17%)	
0 – Not applicable / I don't know	2 (2%)	
**Contact/visit log system**	**3 (2-3)**	**3 (2-3)**
1 – Insufficient	7 (9%)	
2 – Sufficient	30 (37%)	
3 – Good	30 (37%)	
4 – Very good	13 (16%)	
0 – Not applicable / I don't know	2 (2%)	
**Articulation between the three Ministries (Education; Health; and Labor, Solidarity and Social Security)**	**2 (1-3)**	**2 (1-3)**
1 – Insufficient	27 (33%)	
2 – Sufficient	18 (22%)	
3 – Good	24 (29%)	
4 – Very good	10 (12%)	
0 – Not applicable / I don't know	3 (4%)	
**Society perception on early childhood intervention**	**2 (1-3)**	**2 (1-3)**
1 – Insufficient	30 (37%)	
2 – Sufficient	15 (18%)	
3 – Good	23 (28%)	
4 – Very good	10 (12%)	
0 – Not applicable / I don't know	4 (5%)	
**Parents perception on early childhood intervention**	**3 (2-3)**	**3 (2-3)**
1 – Insufficient	19 (23%)	
2 – Sufficient	7 (9%)	
3 – Good	36 (44%)	
4 – Very good	18 (22%)	
0 – Not applicable / I don't know	2 (2%)	
State investment in early childhood intervention	**1 (1-1)**	**1 (1-1)**
1 – Insufficient	60 (73%)	
2 – Sufficient	7 (9%)	
3 – Good	9 (11%)	
4 – Very good	1 (1%)	
0 – Not applicable / I don't know	5 (6%)	

Abbreviations: Md – Median; Q1 – 1st quartile; Q3 – 3rd quartile; 1 – Insufficient; 2- Sufficient; 3 – Good; 4 – Very good. 0- Not applicable / I don't know. § - Values calculated excluding responses with option 0.

Table S4. STROBE Statement—Checklist of items that should be included in reports of observational Studies.

	Item No	Recommendation	Page
**Title and abstract**	1	(*a*) Indicate the study's design with a commonly used term in the title or the abstract	1
		(*b*) Provide in the abstract an informative and balanced summary of what was done and what was found	1
**Introduction**			**2**
Background/rationale	2	Explain the scientific background and rationale for the investigation being reported	2-3
Objectives	3	State specific objectives, including any prespecified hypotheses	3
**Methods**			**3**
Study design	4	Present key elements of study design early in the paper	3
Setting	5	Describe the setting, locations, and relevant dates, including periods of recruitment, exposure, follow-up, and data collection	4
Participants	6	(*a*) Give the eligibility criteria, and the sources and methods of case ascertainment and control selection. Give the rationale for the choice of cases and controls	---
		(*b*) For matched studies, give matching criteria and the number of controls per case	---
Variables	7	Clearly define all outcomes, exposures, predictors, potential confounders, and effect modifiers. Give diagnostic criteria, if applicable	4
Data sources/ measurement	8*	For each variable of interest, give sources of data and details of methods of assessment (measurement). Describe comparability of assessment methods if there is more than one group	4
Bias	9	Describe any efforts to address potential sources of bias	3-4
Study size	10	Explain how the study size was arrived at	3-4
Quantitative variables	11	Explain how quantitative variables were handled in the analyses. If applicable, describe which groupings were chosen and why	4
Statistical methods	12	(*a*) Describe all statistical methods, including those used to control for confounding	4
		(*b*) Describe any methods used to examine subgroups and interactions	3-4
		(*c*) Explain how missing data were addressed	3-4
		(*d*) If applicable, explain how matching of cases and controls was addressed	---
		(*e*) Describe any sensitivity analyses	3-4
**Results**			**5**
Participants	13*	(a) Report numbers of individuals at each stage of study—eg numbers potentially eligible, examined for eligibility, confirmed eligible, included in the study, completing follow-up, and analysed	5
		(b) Give reasons for non-participation at each stage	---
		(c) Consider use of a flow diagram	---
Descriptive data	14*	(a) Give characteristics of study participants (eg demographic, clinical, social) and information on exposures and potential confounders	5-12
		(b) Indicate number of participants with missing data for each variable of interest	5-12
Outcome data	15*	Report numbers in each exposure category, or summary measures of exposure	5-12

Main results	16	(*a*) Give unadjusted estimates and, if applicable, confounder-adjusted estimates and their precision (eg, 95% confidence interval). Make clear which confounders were adjusted for and why they were included	5-12
		(*b*) Report category boundaries when continuous variables were categorized	5-12
		(*c*) If relevant, consider translating estimates of relative risk into absolute risk for a meaningful time period	5-12
Other analyses	17	Report other analyses done—eg analyses of subgroups and interactions, and sensitivity analyses	5-12
**Discussion**			**13**
Key results	18	Summarise key results with reference to study objectives	13-16
Limitations	19	Discuss limitations of the study, taking into account sources of potential bias or imprecision. Discuss both direction and magnitude of any potential bias	16
Interpretation	20	Give a cautious overall interpretation of results considering objectives, limitations, multiplicity of analyses, results from similar studies, and other relevant evidence	13-16
Generalisability	21	Discuss the generalisability (external validity) of the study results	13-16
**Other information**			
Funding	22	Give the source of funding and the role of the funders for the present study and, if applicable, for the original study on which the present article is based	2-3;17

Information on the STROBE is available at http://www.strobe-statement.org.
